# Stent-in-Stent Technique for the Treatment of Proximal Bronchial Restenosis after Insertion of Metallic Stents: A Report of Two Cases

**DOI:** 10.1155/2016/6742349

**Published:** 2016-03-24

**Authors:** Benjamin Bondue, Pascal Schlossmacher, Christiane Knoop, Isabelle Etienne, Sylvie Luce, Youri Sokolow, Dimitri Leduc

**Affiliations:** ^1^Department of Chest Medicine, Erasme University Hospital, Free University of Brussels, 1070 Brussels, Belgium; ^2^Department of Chest Medicine, La Reunion University Hospital, Saint Denis, 97400 La Réunion, France; ^3^Department of Medical Oncology, Erasme University Hospital, Free University of Brussels, 1070 Brussels, Belgium; ^4^Department of Thoracic Surgery, Erasme University Hospital, Free University of Brussels, 1070 Brussels, Belgium

## Abstract

Endoscopic treatment of a bronchial restenosis previously treated by insertion of a partially covered self-expandable metallic stent (SEMS) can be difficult. Classically, after recanalization of the bronchus, the stent is removed and replaced by a more adapted one. We report on two cases of proximal bronchial restenosis treated by insertion of an additional stent inside the lumen of the previously inserted stent using the stent-in-stent (SIS) technique. The indications for the initial stent were malignancy in Patient 1 and posttransplant bronchial stenosis in Patient 2. Restenosis occurred at the proximal end of the stent within months in both cases. Stent removal and insertion of a new stent were considered, but this option was discarded because of an excessive risk of bronchial perforation and preference towards an alternative approach. In both cases, a second customized SEMS was placed using the SIS technique after ablation of the proximal end stenosis of the stent by argon plasma coagulation and/or dilation with a balloon. Recanalization of the bronchus was achieved in both cases without complications. The SIS technique is a valuable alternative to removal of SEMS in case of proximal bronchial restenosis.

## 1. Introduction

Insertion of bronchial SEMS is a classical treatment for bronchial stenosis, especially for malignant and posttransplant stenosis [1–3]. Covered SEMS are useful to avoid the risk of intrastent stenosis (between the extremities), but restenosis can still occur at both ends. After lung transplantation, 25% of SEMS have to be removed due to excessive granulation tissue formation and stent obstruction [4]. In malignancies, restenosis is related to proliferation of either malignant or granulation tissues. Recanalization can be performed using laser, argon plasma coagulation, balloon dilation, or cryotherapy. Removal of the stent and its replacement by a longer stent can be indicated. However, removal of metallic stent can be technically challenging with a risk of bronchial perforation and bleeding, especially if the stent is not totally covered and is anchored into the bronchial mucosa.

The SIS technique is described in some gastroenterological indications (e.g., endoscopic biliary drainage) [5] and consists in inserting a new stent into the lumen of the stent already in place. However, only few reports describe the use of the SIS technique in interventional bronchoscopy [6, 7]. Of note, none concerned lung transplant recipients.

We report on two cases, including one after lung transplantation, presenting a proximal bronchial restenosis successfully treated by the SIS technique.

## 2. Case Report #1

**Figure 1:**
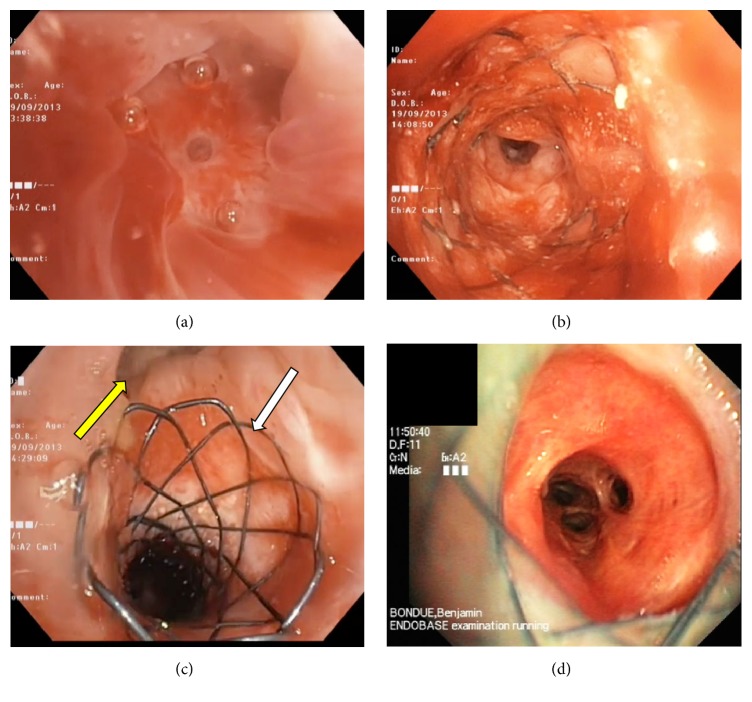
(a) Endoscopic view of the near-complete posttransplant restenosis of the right bronchus intermedius. A small residual orifice allows a recanalization by forceps first and then balloon dilation and argon plasma therapy. (b) Endoscopic view of the previously inserted metallic stent in the right bronchus intermedius below the proximal stenosis. The distal end is patent but anchored in a highly inflammatory tissue. (c) View of the proximal end of the newly inserted stent using the stent-in-stent technique. Note the spherical end with a higher diameter (10 mm instead of 8 mm, white arrow) and the patent right upper bronchus (yellow arrow). (d) Endoscopic view of the distal end of the prosthesis 1 month later. The right lower lobe is ventilated and inflammation is resolved.

A 60-year-old woman was transplanted in 2011 for severe emphysema. She developed a posttransplant stenosis of the right bronchus intermedius, which required the insertion of a partially covered SEMS (diameter 8 mm, length 20 mm). However, a restenosis of the right bronchus intermedius occurred 5 months later because of proliferation of fibrous and granulation tissue at the proximal end of the SEMS (Figure 1(a)). The restenosis required multiple recanalization procedures using argon plasma coagulation and balloon dilation (*n* = 5, mean interval of 2,5 months between each intervention). Below the proximal obstruction, both uncovered ends of the stent appeared deeply anchored into the bronchial mucosa (Figure 1(b)) while the distal end opened onto a patent right lower lobe bronchus. It appeared technically difficult to remove the stent without a significant risk of bronchial perforation or bleeding. Therefore, a second SEMS (25 × 8 mm, Microtech) was designed according to the stricture characteristics. The proximal end of this stent was uncovered and spherical to increase its diameter (10 mm instead of 8 mm (Figure 1(c))) and, thus, to reduce the risk of migration. In contrast, the distal end was cylindrical and fully covered to facilitate insertion into the previously placed stent. The second stent was successfully inserted into the first one under fluoroscopic guidance using the SIS technique allowing for reventilation of the right lower lobe (Figure 1(d)). Lung function improved significantly after the intervention with an increase of 800 mL of both vital capacity (VC) and forced expiratory volume in one second (FEV1). No restenosis occurred with an endoscopic follow-up of 2 years.

## 3. Case Report #2

**Figure 2:**
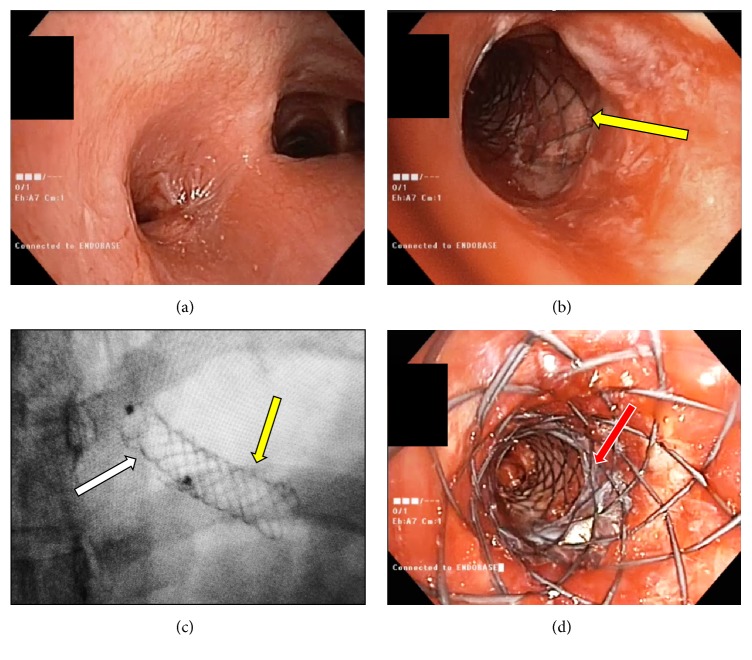
(a) Endoscopic view of the left main bronchus stenosis. The stenosis was of a mixed origin: malignant and postradiotherapy fibrotic stricture. (b) After recanalization of the main left bronchus, the previously inserted stent is visualized (yellow arrow). (c) Fluoroscopic view of the two stents positioned in the left main bronchus: the proximal and newly inserted stent (white arrow) and the previous distal stent (yellow arrow). (d) Final endoscopic view showing the junction between the two stents (red arrow).

A 32-year-old woman with an adenoid cystic carcinoma of the left upper lobe developed an almost complete stenosis of the left main bronchus and the left upper lobe bronchus. Initially, a partially covered SEMS was inserted into the main left bronchus with a distal opening into the left lower lobe bronchus (length 30 mm, diameter 8 mm). Three weeks after initial intervention a complete restenosis occurred at the proximal end due to proliferation of tumoral tissue. Radiochemotherapy led to a partial response of the tumor but also to a fibrotic stenosis of the left main bronchus (Figure 2(a)). A small 2 mm diameter opening allowed for a recanalization of the left main bronchus by successive balloon dilations (8 mm diameter). As the initial stent was firmly anchored into the mucosa and the bronchus distorted by the tumor and postradiation fibrosis, a removal of the prosthesis was deemed impossible (Figure 2(b)). Therefore, a custom-made partially covered stent (8 mm diameter, Microtech) was successfully inserted into the first one under fluoroscopic guidance using a wire (Figures 2(c) and 2(d)). Only the proximal end was uncovered and spherical to increase the diameter to 10 mm and reduce the risk of migration. After an endoscopic follow-up of 2 months, the second stent is still in place and the left lower lobe bronchus is still patent.

## 4. Discussion

Bronchial stenosis results from malignant or nonmalignant causes, which include sarcoidosis, tuberculosis, Wegener's granulomatosis, and iatrogenic conditions, such as postsurgical anastomosis and radiation therapy as the most frequent etiologies [8, 9].

Treatment options include dilation of the stenotic airway with a balloon or debulking with a bronchoscope, laser therapy, argon plasma coagulation, electrocautery, cryotherapy, and insertion of silicone or self-expandable metallic stents (SEMS) [8]. Silicone stents are used in both malignant and benign conditions whereas SEMS are used for the treatment of malignancies but avoided for benign airway disorders especially for totally uncovered SEMS. Commercially available SEMS are totally covered, partially covered or uncovered. Covered SEMS are used to prevent tumor ingrowth for malignant airway obstruction. Compared to silicone stents, SEMS are easily placed, have a larger internal/external diameter ratio, and require less mechanical pressure on the bronchial wall during their insertion. These two latter characteristics justify their cautious use in our center and by others for the treatment of benign posttransplant stenosis [4, 9, 10], especially when there is a risk of bronchial breaking.

Indeed, SEMS are effective in the immediate management of posttransplant bronchial stenosis but have a high complication rate. The optimal treatment in this setting is unknown. Silicone stents are an alternative [11] but their use in posttransplant stenosis is also limited because lesions are often complex and have a smaller inner diameter and there is a potential risk of bronchial breaking during the insertion of the silicone stent through recent anastomosis. Complications of airway stenting with SEMS include restenosis, migration, and bronchial rupture. Among lung transplant recipients, the rate of restenosis ranges between 25% and 53% and may occur early or late (>30 months after insertion) [4, 9]. Therefore, the use of SEMS in the management of posttransplant stenosis should be carefully discussed and restricted when no other option is available. Moreover, a close bronchoscopic follow-up is recommended, especially during the first year after insertion [9, 12]. In malignancies, a rate of restenosis of 18% is described with SEMS [13]. In many cases, a SEMS can be removed and replaced [4, 13]; however, when the SEMS is embedded in the bronchial mucosa or fractured its removal may become extraordinarily difficult [8, 14, 15]. In case of embedded stent, an oversized silicone stent can be inserted into the SEMS leading to necrosis of the bronchial mucosa allowing for the subsequent removal of the stent [15]. This technique was, however, not conceivable in our 2 cases because of the small diameter of the SEMS (8 mm), the minimal required diameter for the insertion of a silicone stent being 10 mm. We thus resorted to the SIS technique in both cases. This technique is often used by gastroenterologists, especially for the management of biliary stenosis [5]. In these indications, a success rate of 80%–100% is reported with a patency period ranging between 140 and 238 days [5]. In interventional bronchoscopy, the use of this technique is seldom reported [6, 7]. Watanabe et al. reported its use in a case of a malignant tracheal restenosis 1 year after the placement of a metallic stent [6]. Xu et al. demonstrated that Gianturco-Z stents can be used with a SIS technique for the palliation of (mostly malignant) tracheobronchial stenosis and contribute to improving the quality of life in patients with advanced cancer [7]. To our knowledge, it is the first report of the use of this technique in a lung transplant recipient.

In conclusion, the SIS technique is feasible and safe to treat proximal bronchial restenosis of a previously inserted stent. It is a valuable alternative if the removal of the previously inserted stent is deemed too difficult and/or if the insertion of an oversized silicone stent is impossible. It is useful not only in malignant conditions, but also in case of posttransplant bronchial stenosis. This approach may prove to be a valuable alternative in various clinical scenario and etiologies including malignant and benign conditions.
